# A Barcoded Flow Cytometric Assay to Explore the Antibody Responses Against SARS-CoV-2 Spike and Its Variants

**DOI:** 10.3389/fimmu.2021.730766

**Published:** 2021-09-23

**Authors:** Niklas Vesper, Yaneth Ortiz, Frauke Bartels-Burgahn, Jianying Yang, Kathrin de la Rosa, Matthias Tenbusch, Sebastian Schulz, Stephanie Finzel, Hans-Martin Jäck, Hermann Eibel, Reinhard E. Voll, Michael Reth

**Affiliations:** ^1^ Institute of Biology III, Faculty of Biology, University of Freiburg, Freiburg, Germany; ^2^ Research Centres Bioss, Centre for Biological signal studies, CIBSS, Centre for Integrative Biological Signalling Studies, University of Freiburg, Freiburg, Germany; ^3^ Department of Cancer and Immunology, Max-Delbrück-Center for Molecular Medicine in the Helmholtz Association (MDC), Berlin, Germany; ^4^ Institute of Clinical and Molecular Virology, University Hospital Erlangen, Friedrich-Alexander University Erlangen-Nuremberg, Erlangen, Germany; ^5^ Division of Molecular Immunology, Internal Medicine III, Nikolaus-Fiebiger-Center of Molecular Medicine, Friedrich-Alexander University Erlangen-Nuremberg, Erlangen, Germany; ^6^ Department of Rheumatology and Clinical Immunology, Medical Center – University of Freiburg, Faculty of Medicine, University of Freiburg, Freiburg, Germany; ^7^ Center for Chronic Immunodeficiency, Medical Center, University of Freiburg, Freiburg, Germany

**Keywords:** COVID-19, coronavirus, SARS-CoV-2, virus variants, spike protein, RBD, humoral immunity, flow cytometry

## Abstract

The SARS-CoV-2 pandemic has spread to all parts of the world and can cause life-threatening pneumonia and other severe disease manifestations known as COVID-19. This health crisis has resulted in a significant effort to stop the spread of this new coronavirus. However, while propagating itself in the human population, the virus accumulates mutations and generates new variants with increased fitness and the ability to escape the human immune response. Here we describe a color-based barcoded spike flow cytometric assay (BSFA) that is particularly useful to evaluate and directly compare the humoral immune response directed against either wild type (WT) or mutant spike (S) proteins or the receptor-binding domains (RBD) of SARS-CoV-2. This assay employs the human B lymphoma cell line Ramos, transfected for stable expression of WT or mutant S proteins or a chimeric RBD-CD8 fusion protein. We find that the alpha and beta mutants are more stably expressed than the WT S protein on the Ramos B cell surface and/or bind with higher affinity to the viral entry receptor ACE2. However, we find a reduce expression of the chimeric RBD-CD8 carrying the point mutation N501Y and E484K characteristic for the alpha and beta variant, respectively. The comparison of the humoral immune response of 12 vaccinated probands with 12 COVID-19 patients shows that after the boost, the S-specific IgG class immune response in the vaccinated group is similar to that of the patient group. However, in comparison to WT the specific IgG serum antibodies bind less well to the alpha variant and only poorly to the beta variant S protein. This is in line with the notion that the beta variant is an immune escape variant of SARS-CoV-2. The IgA class immune response was more variable than the IgG response and higher in the COVID-19 patients than in the vaccinated group. In summary, we think that our BSFA represents a useful tool to evaluate the humoral immunity against emerging variants of SARS-CoV-2 and to analyze new vaccination protocols against these variants.

## Introduction

Since December 2019, the severe acute respiratory syndrome coronavirus 2 (SARS-CoV2) has been spreading in the human population as a pathogen causing the coronavirus disease 2019 (COVID-19) associated with severe pneumonia (1). The rapid spread of this pandemic virus and the severity of this worldwide health crisis is associated with three features (2). First, SARS-CoV-2 is a new member of the beta-coronavirus family and hence there is no human immunity against this emerging virus (3). Second, SARS-CoV-2 enters the cells *via* the angiotensin-converting enzyme 2 (ACE2), a receptor widely expressed in human mucosal tissues of the nose and mouth and particularly abundant in the lung (4, 5). Third, SARS-CoV-2 is a positive-strand RNA virus and can thus rapidly generate mutations (6). The virus binds to the ACE2 entry receptor *via* the trimeric spike (S) protein prominently expressed on the viral membrane (7). Upon binding to ACE2, the S protein undergoes a conformational change. It is cleaved by cellular proteases into an S1 and S2 portion, with the latter inducing a fusion reaction between the viral and cellular membrane, thereby starting the infection cycle (8). The RBD, that directly binds to ACE2 is located within the S1 portion. The structure of the SARS-CoV-2 trimeric S protein has been determined by cryo-electron microscopy at the atomic level. It has revealed that the RBD can assume a closed (down) or open (up) conformation, with only the latter being able to interact with the ACE2 entry receptor (9).

The trimeric S protein of SARS-CoV-2 is a prominent target of the humoral immune response (9–11). In particular, RBD-specific antibodies can inhibit the binding of the S protein to ACE2 and thus function as neutralizing antibodies that block viral entry into the target cells (4). Indeed, it has been found that the RBD is an immunodominat structure of the S protein and targeted by more than 90% of the neutralizing antibodies (12). Several specific monoclonal antibodies (mAb) have been generated and are used in the clinic as therapeutic reagents to treat acute COVID-19. These mAb can be directed to the full-length S protein or the RBD domain. A co-crystallization of Fab fragments of anti-RBD mAb with the RBD resulted in their classification in ACE2-blocking or non-blocking antibodies (9, 12). The S protein or its encoding mRNA are used for the rapid development of vaccines that counteract the spread of the SARS-CoV-2. In particular, mRNA vaccines could be rapidly produced and play an essential role in the worldwide vaccination programs counteracting the spread of the virus. However, these efforts may be compromised by the appearance of SARS-CoV-2 variants that start to spread in different parts of the world (6). The SARS-CoV-2 variants carry characteristic mutations in the RBD and other parts of the S protein. According to the World Health Organization’s recommendations (WHO), they are now classified as Greek letters (13). They are usually characterized by their increased infectivity, their ability to multiply more rapidly in an infected host and to escape recognition by at least some neutralizing antibodies generated against the S protein of WT SARS-CoV-2. Hence, the virus variants represent “fitness” and/or “immune evasion” mutants (14).

Many S protein-based serological assays have been developed to evaluate the success of the diverse vaccination programs and determine the anti-SARS-CoV-2 humoral immune status of a human population. These are based on linear peptides or the full-length S protein and frequently use the ELISA technique (15). Recently also flow cytometric techniques have been introduced (16–18). The advantage of these assays is that they use cells expressing native S proteins with the same orientation and a glycosylation pattern similar to that found on the viral membrane. This also applies to a cell-based enzyme-linked immunosorbent assay used for the detection of a HIV or SARS-CoV-2 infection (19, 20). Here we describe a flow cytometric assay that allows comparison of the humoral immune response of vaccinated and infected persons in terms of its reactivity towards the S protein and RBD of either WT or the variants of SARS-CoV-2.

## Materials and Methods

### Participants

Four female and eight male COVID-19 patients (n=12) with mean ages of 65 and 62 years were recruited at the Medical Center University of Freiburg. Written informed consent was obtained from all participants in this study. Convalescent plasma was collected according to the FDA recommendation. Donors met routine FDA-established blood donor eligibility requirements and had previous SARS-CoV-2 infection documented by laboratory testing for the virus during illness or antibodies to the virus after recovery from suspected illness. In addition, six female and six male subjects (n=12) vaccinated with BioNTech/Pfizer Comirnaty were recruited with a mean age of 49.33 and 44.83 years, respectively at the Medical Center – University of Freiburg. Written informed consent was obtained from all participants. The study was approved by the Ethical Committee of the University of Freiburg (EK-Freiburg no 315/10). Serum and plasma samples were aliquoted and stored at−80°C.

### Cloning

The retroviral expression vectors encoding the WT, alpha or beta variant S protein of SARS-CoV-2 are based on the pMIG vector backbone (pMIG was a gift from William Hahn [Addgene plasmid # 9044; http://n2t.net/addgene:9044; RRID: Addgene_9044)]. The cDNA of the relevant S protein genes was amplified by PCR with primers containing the proper extensions to be ligated into the linearized pMIG vector by a Gibson assembly-like method, namely the In-Fusion cloning protocol from ClonTech. To connect the PCR fragments, we designed them so that they have a 15 base pair overlap. The cDNA encoding the WT S protein is derived from the plasmid pVAX1-SARS2-S with a codon-optimized sequence of the S protein gene of SARS-CoV-2 (21). The alpha and beta S protein cDNA were synthesized in ITD gBlocks. The retroviral expression vector encoding the RBD-CD8 chimeric const ruct is based on the pMIG-CD8 vector containing the murine CD8 gene cDNA. The cDNA encoding the RBD of the WT S protein was amplified by PCR and ligated into the linearized pMIG-CD8 vector so that the RBD was placed in between the leader peptide and the extracellular Ig domain of CD8. The point mutations in the RBD-CD8 construct were generated by site-directed mutagenesis. For the design of primers and plasmids, we used Geneious 9.0.5 software. The component mixture used for the PCR and the PCR program was set up according to CloneAmp HIFI PCR Premix. All generated vectors were sequenced (Eurofins Genomics) and the sequencing results were analyzed by Geneious software

### Cell Culture

Ramos B cells were cultured in RPMI medium supplemented with 10% fetal bovine serum (FBS), 1% penicillin-streptomycin and 0.12% β-mercaptoethanol (RPMI+). The Ramos B cell cultures were split every 2 days.

### Retroviral Production in Phoenix Cells and Transduction of Ramos B Cells

Phoenix cells were cultured in iscove basal medium (IBM) supplemented with 10% FBS and 1% penicillin streptomycin (IBM+). Cells were split every 2 days by diluting them 1/10. One day before the transfection with the retrovirus producing plasmids pKAT and pMIG, 5x10^5^ Phoenix cells at 70 % confluency were pipetted into a coated 6-well plate. Afterward, the cells were supplemented with 2 ml of IBM+. Between 18 and 24 hours later, the transfection was performed on cells at 70% confluency, using Polyjet transfection reagent, according to the manufacturer’s instructions. After 2 days of culture, the virus-containing supernatant was collected and filtered through a 0.45 µm filter. Afterward, Polybrene was added at a concentration of 1 µl/ml. Ramos-null B cells were split the day before the transduction, 6x10^5^ Ramos-null cells were resuspended in 1 ml of the transduction mixture and then centrifuged for 3 h at 400xg and 37°C. After this step, the viral supernatant was replaced with RPMI+. The cells were then transferred to a 12-well plate in a total volume of 2.5 ml of medium.

### Barcoding

For barcoding, 10^6^ Ramos B cells were resuspenden in 1 ml of DPBS alone or with different concentrations of the cell proliferation tracer CytoTell blue (1:250, 1:1250, 1:10.000). The cells were incubated for 30 min in the dark at room temperature, washed twice with DPBS and then combined in one test tube for further staining.

### Cell Surface Staining and Subsequent Analysis

The following flow cytometry antibodies were obtained from BioLegend: anti human IgG (M1310G05), anti human IgA (HP6123), anti mouse CD8 (53-6.7), anti mouse kappa light chain (RMK-12). For primary staining, barcoded cells were incubated with the binding reagent for 30 min at 4 °C in the dark. After incubation, the cells were washed twice with DPBS. Secondary staining was performed under the same conditions using appropriate fluorescent label secondary antibodies. The cells were washed resuspended in DPBS for FACS analysis on a ThermoFisher Attune NxT flow cytometer. Filters used 440/50, 530/30, 585/16 and 670/14. Flow cytometry data were analyzed with FlowJo v10 (Tristart). The mean fluorescent intensity (MFI) values from each cell line were then normalized to the MFI from the control Null cell line within each barcoded sample, and the resulting normalized MFI was used for comparison of binding to different Spikes or RBDs variants.

### ACE-Ig Reagent and mAb

ACE2-Ig was cloned by fusing the human *ACE2* Q18-V739 fragment to the human IgG1-Fc portion (E99-K330 portion, where 1st amino acid is G encoded by J-CH1 fusion) of the expression vector from Oxford Genetics (pSF-CMV-HuIgG1). Cloning constructs were used to transfect FreeStyle 293-F cells that were grown in suspension using FreeStyle 293 expression medium (Life Technologies) at 37°C in a humidified 8% CO2 incubator rotating at 125 rpm. Cells were grown to a density of 2.5 million cells per mL, transfected using PEI (4 µg/mL in cell suspension) and DNA (1200 ng/ml in cell suspension), and cultivated for 3 days. The supernatants were harvested and ACE2-Ig was purified by protein G SpinTrap columns according to manufacturer’s instructions (Cytiva, 28903134).

The isolation of monoclonal TRES antibodies is described in (22). Briefly, TRIANNI C57/Bl6 mouse line HHKKLL (Patent US 2013/0219535 A1) was primed with a plasmid encoding wild type SARS-COV-2 spike protein and boosted twice intramuscularly with stabilized trimeric S protein of SARS-CoV-2 adjuvanted with Monophosphoryl Lipid A (MPLA) liposomes (Polymun Scientific GmbH, Klosterneuburg, Austria). Spleen cells were fused with Sp2/0 cells, and hybridoma clones were screened for spike-binding antibodies with a flow-based assay as described by (17). Positive clones were subcloned by the limiting dilution method. Rearranged VH and VL exons were cloned by the 5’ Race method and sequenced.

### Anti-SARS-CoV-2-ELISA IgG

Sera of vaccinated persons, diluted 1/100, were tested for anti-spike IgG antibodies by ELISA, using the EI 2601-9601 G SARS-CoV-2 Spike IgG kit from Euroimmun applying the reagents and the protocol provided by the manufacturer. The CE certified assay is widely used to determine the presence of SARC-COV-2 specific antibodies (23). According to the manufacturer, the assay’s sensitivity is 94,4% and the specificity 99.8%

## Results

### Setting Up the Spike Flow Cytometric Assay

**Figure 1:**
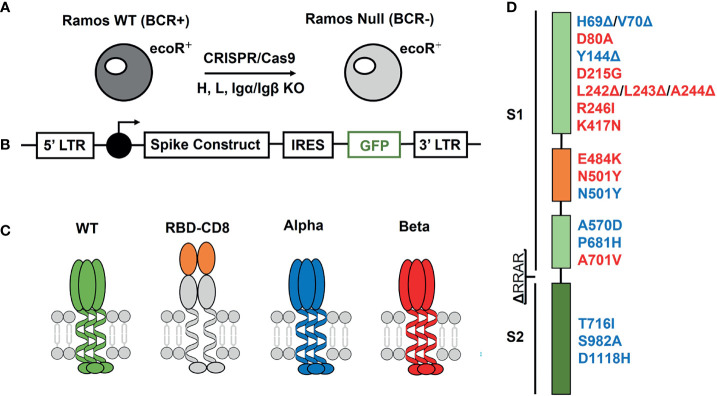
Expression system for the generation of Ramos-null B cells carrying on their surface WT or variant spike proteins of SARS-CoV-2. **(A)** Generation of the Ramos-null cells lacking functional genes for the four components (heavy chain, light chain, Igα, Igβ) of the IgM-BCR by the CRISPR/Cas9 technique. These cells also carry the ecoR receptor for efficient retroviral transfection. **(B)** Retroviral vector used for the linked expression of the spike protein and GFP using an internal ribosome entry site (IRES) sequence in front of the GFP cDNA. **(C)** Schematic drawing of the expressed S proteins with the WT (green), the receptor binding domain (RBD) (orange) attached to the murine CD8 protein (grey), the alpha variant B.1.1.7 (blue) and the beta variant B.1.3.5.1 (red). **(D)** Location of the amino acid exchange or deletion mutations in the S1 and S2 domain of the S protein. Alterations in the S protein of the alpha variant are indicated in blue and those of the beta variant in red.

The human Burkitt lymphoma cell line Ramos is a valuale tool of immunological research (24). These cells can be propagated efficiently in cell cultures and modified by the CRISPR/Cas9 technique (25). In our study, we used a Ramos-null line lacking all four components of the B cell antigen receptor (BCR), namely heavy chain, light chain, Igα and Igβ (26). Although the Ramos-null cells grow more slowly than Ramos wild type (WT), they can be maintained in culture and expanded to large cell numbers. Furthermore, the Ramos-null cells also carry on their surface a receptor for ecotropic retroviruses (ecoR) that allows the efficient transduction of these cells with murine retroviral vectors (**Figure 1A**). For the expression of different S constructs on the surface of Ramos-null cells, we used a pMIG vector carrying after the LTR promoter the construct sequence, an internal ribosome entry site (IRES) and the sequence coding for the green fluorescent protein (GFP) (**Figure 1B**). This vector system allows us to detect and enrich the transduced (GFP-positive) Ramos-null cells and correlate to some extent the expression of the S protein with that of GFP. The different retroviral vectors were used to express either the S protein of SARS-CoV-2 or a chimeric protein carrying the isolated RBD of the S protein in front of the murine CD8 molecule.

Furthermore, we also expressed the S protein of two SARS-CoV-2 mutants, namely the alpha variant (B1.1.7) and the beta variant (B.1.351) (**Figure 1C**). These mutant S proteins differ from the WT SARS-CoV-2 sequence at 8-10 amino-acid positions (**Figure 1D**). In the RBD sequence, the mutant S proteins carry critical amino acid exchange mutations that influence the binding of the S protein to the ACE2 target and its detection by neutralizing antibodies (6, 27). These are an asparagine to tyrosine exchange (N501Y) in the alpha variant and the same mutation in combination with a glutamate to lysine (E484K) exchange mutation in the beta variant. In addition, the S proteins expressed on the Ramos cell surface carry a deletion of four amino acids (RRAR) at the border between the S1 and S2 domains, preventing their cleavage by cellular proteases.

**Figure 2:**
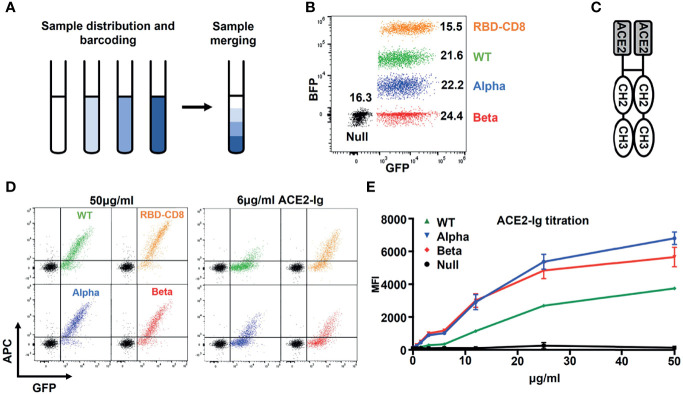
Establishment of the color-based barcoded spike flow assay (BSFA). **(A)** Labeling of four different Ramos cell populations by exposure to different concentrations (0, 1:250, 1:1250, 1:10.000) of *CytoTell* blue for further analysis in one test tube. **(B)** Gating strategy for the analysis of four different Ramos cell populations by flow cytometry. The *CytoTell* blue -loaded Ramos cells are separated by the blue fluorescent protein (BFP) gate. **(C)** Structural model of the chimeric ACE2-Ig molecule used for the detection of the S protein or the RBD-CD8 chimera on transfected Ramos cells. **(D)** Analysis of Ramos cells expressing the WT (green), alpha variant (blue), beta variant (red) S protein or the RBD-CD8 construct (orange). The cells were exposed to either 50 or 6 ug/ml of ACE2-Ig, washed, stained with APC-coupled anti-human IgG antibodies and then analyzed by flow cytometry. Shown is the dot plot depicting the GFP and APC fluorescence form each one of the variants overlay with the Ramos-Null (GFP-) control cell line. **(E)** Titration of the ACE2-Ig binding to the four indicated Ramos cell populations. As negative control we used Ramos cells without S protein (black). The mean values of three different experiments are shown.

To directly compare Ramos cells expressing different S protein constructs in their binding of either a soluble ACE2 or anti-SARS-CoV-2 antibodies, we developed a color-based barcoded spike protein flow cytometric assay (BSFA). To this end, we first incubated Ramos cells with PBS alone or with different concentrations of the cell proliferation tracer *CytoTell* blue. The Ramos cells are then washed and combined in one sample tube (**Figure 2A**) before being stained with different binding reagents. Finally, the combined Ramos cells are separated by a flow cytometric gate using the blue fluorescent protein (BFP) channel (**Figure 2B**). To test for the expression of the different S constructs, we used an ACE2-immunoglobulin (ACE2-Ig) chimeric protein carrying at the C-terminus instead of the TM region of ACE2 the CH2 and CH3 domains of human IgG1 (**Figure 2C**). The different Ramos cells were first incubated with increasing concentrations of ACE2-Ig, washed, stained with allophycocyanin (APC)-coupled anti-human IgG antibodies and then analyzed for APC and GFP fluorescence by flow cytometry (**Figure 2D**). We found that Ramos cells expressing the RBD-CD8 construct show the most robust ACE2-Ig binding. This is in line with a structural analysis showing that only a minority of the trimeric S proteins display the RBD in an open (ACE2-binding) conformation (9, 28), whereas as part of the RBD-CD8 construct, the RBD should be fully accessible for ACE2 binding. The directed comparison of Ramos cells expressing the WT S constructs or the alpha and beta variants shows that the variants display a stronger ACE2-Ig binding (**Figure 2D**). This is particularly visible at the lower (6 ug/ml) concentration of ACE2-Ig and confirmed by a more detailed titration experiment (**Figure 2E**). These data show that the BSFA can be used to evaluate the expression and binding activity of WT and mutant SARS-CoV-2 S proteins to ACE2.

### Evaluation of Anti-S Antibodies and Sera

**Figure 3:**
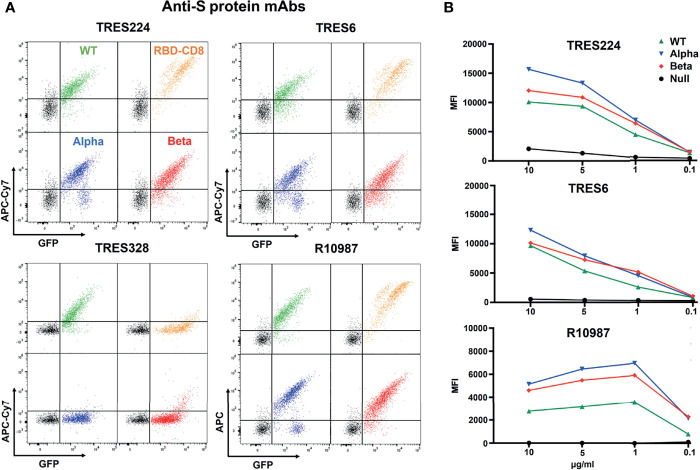
BSFA study of the binding of four monoclonal antibodies (mAb) to S protein- expressing Ramos cells. **(A)** Ramos cells carrying the WT (green), alpha variant (blue), beta variant (red) S protein or the RBD-CD8 construct (orange) cells were incubated with 1 ug/ml of the mAb TRES224, TRES6, TRES328 and R10987. After washing, the cells were stained with either APC-Cy7-coupled anti-mouse IgG antibodies (for TRES224, TRES6, TRES328) or APC-coupled anti-human IgG antibodies (for R10987) and analyzed for the APC-Cy7/APC and GFP color by flow cytometry. **(B)** Titration of the mAb TRES224, TRES6 and R10987 to Ramos cells carrying the indicated S proteins. As negative control we used Ramos cells without S protein (black). The mean values of three different experiments are shown.

We next incubated the four different Ramos cell lines with 1 ug/ml of monoclonal antibodies (mAb) generated against the WT trimeric S protein of SARS-CoV-2. These TRIANNI-Erlangen anti-SARS-CoV-2- Spike (TRES) mAb are either directed against the hACE2 binding site (TRES224, TRES6) or the N-terminal domain (TRES328) of the S protein (22). The BSFA showed that the TRES224 and TRES6 antibodies are indeed directed against the RBD of the S protein whereas TRES328 hardly binds this structure (**Figure 3A**). A strong RBD binding was also found when using the therapeutic anti-SARS-Cov-2 antibody R10987 characterized as a class 3 RBD binder (9). The RBD-specific mAb TRES224, TRES6 and R10987 also bind well to the alpha and beta variant S proteins, which is not true for TRES328. Interestingly, the titration of the three RBD-specific mAb shows that they bind even better to Ramos cells expressing the variant than those expressing the WT S proteins (**Figure 3B**). These data demonstrate that on the Ramos cell surface the variant S proteins are expressed as well as or even better than the WT S protein and that the BSFA can be used to evaluate the fine specificity of anti-SARS-CoV-2 antibody response.

**Figure 4:**
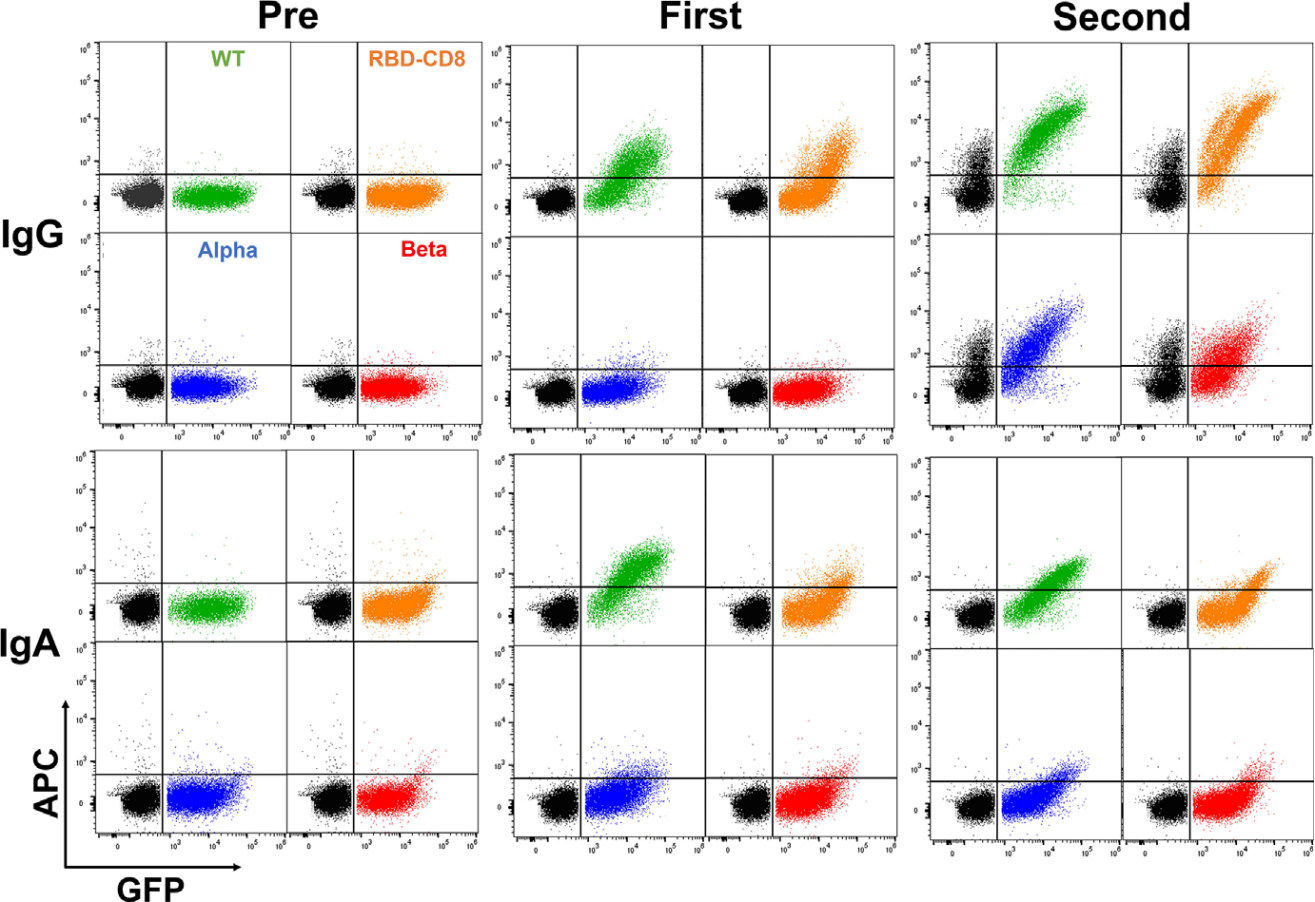
BSFA study of the serum of a vaccinated (BioNTech/Pfizer) 47-year-old female (V7) for anti-S protein IgG (upper panels) and IgA class (lower panels) antibodies. Serum was collected 1 day before (pre) or 12 days after the first vaccination and 10 days after the second vaccination.

Having demonstrated that the BSFA works well in the evaluation and characterization of anti-S antibodies, we next analyzed sera from 12 persons (V1-V12) who had been vaccinated with the BioNTech/Pfizer mRNA vaccine (Comirnaty^®^). For each person, we obtained sera before or shortly after vaccination, 10-14 days after the first and 10-15 days after the second vaccination (**Table 1**). As an example, for the human humoral response after vaccination, we show a BSFA study for S-specific IgG and IgA antibodies in the sera of a 47-year-old female (V7) taken either 1 day before or 12 days after the first and 10 days after the secondary (boost) vaccination (**Figure 4**). No S-specific antibodies were found before immunization, and that was the case for all analyzed sera (**Supplementary Figure 1**). After the first vaccination, the serum of V7 contained specific IgG antibodies directed against the WT and RBD-CD8 but not against the alpha and beta variant S proteins. The primary IgA antibody response of V7 is also predominantly directed towards the WT but not the variant S proteins. After the secondary immunization, the amount of IgG antibodies directed against the WT S protein and RBD has increased. The sera now also contain IgG antibodies binding to the alpha and beta variant S proteins. However, in contrast to the previously tested mAb, the serum antibodies of V7 recognize the WT better than the alpha and beta variants’ S proteins. (**Figure 4**, outer right panel). The secondary IgA antibody response did not improve much compared to the primary response. Only a few IgA antibodies bound to the Ramos cells expressing high amounts of the alpha and beta variants’ S proteins.

**Table 1:** Demographic data of SARS-CoV-2 infected patients.

Patient cohort	Days between diagnose and sampling	Sex	Age
C1	51	M	59
C2	94	M	53
C3	39	M	67
C4	28	M	61
C5	19	F	78
C6	8	M	74
C7	48	F	66
C8	34	F	50
C9	26	F	68
C10	46	M	65
C11	16	M	63
C12	19	M	56

Median age: 64+/- 8,18 years; M, male; F, female.

**Figure 5:**
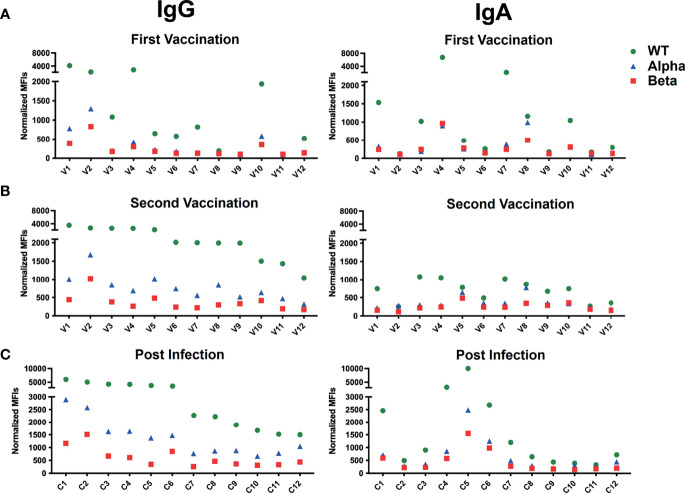
BSFA study of the sera of 12 persons vaccinated with the BioNTech/Pfizer mRNA vaccine after the primary **(A)** and secondary **(B)** vaccine doses and of 12 patients with severe COVID-19 disease **(C)**. Summary of the production of S protein-specific IgG (left panel) or IgA (right panel) antibodies. The sera were diluted 1:100 and analyzed by flow cytometry for antibodies binding to Ramos cells expressing either the WT (green), the alpha variant (blue) or the beta variant (red) S protein. Shown are normalized mean fluorescence intensity (MFI).

The analysis of sera from all 12 vaccinated persons confirms these findings (**Figures 5A, B**). The S-specific IgG antibody response is in most cases higher in the secondary than in the primary sera. The secondary sera also contain IgG antibodies against the alpha and beta variants S proteins, albeit at a lower level. This is in line with a study showing that the protective antibody respose of mRNA vaccinated person is sufficient but lower in the case of the alpha mutant (29). Four of the 12 vaccinated persons (V1, V2, V4 and V10) already had a high anti-S primary IgG response that did not improve substantially after the secondary immunization. The S-specific IgA antibody response is more variable from person to person and is not always improved in the secondary response. Most sera of the vaccinated group had IgA antibodies that bind more strongly to Ramos cells expressing the WT than those expressing the alpha and beta variants’ S proteins. For comparison we also studied the blood samples of the 12 vaccinated persons with an ELISA detecting IgG antibodies directed against the S1 part of the WT S protein (**Supplementary Figure 2**). This assay also shows that in most cases the IgG antibody response is higher in the secondary than in the primary response. However, there are some discrepancies in the variability of the primary response and the secondary response of V7 that may be due to the detection of different epitopes on the isolated S1 part of the spike on plastic *versus* full-length spike on the plasma membrane by the anti-SARS-CoV-2 antibodies. Nonetheless, there is an overall good correlation between the ELISA and the BSFA which strengthens the validity of the flow cytometric assay.

We next analyzed the blood of 12 COVID-19 patients (C1-C12) (**Table 2**). These sera were taken 1-12 weeks after the SARS-CoV-2 infection and were analyzed by BSFA for S-specific IgG or IgA antibodies (**Figure 5C**). All 12 persons developed S-specific IgG class antibodies. The 12 tested persons could be grouped into 6 high and 6 low responders with a normalized MFI of above 5000 and below 3000, respectively. The sera of all infected persons also had IgG antibodies that bound to Ramos cells expressing the alpha and beta variant S proteins, albeit with lower binding intensity. The specific IgG antibodies always bound Ramos cells with the alpha variant better than those carrying the beta variant. This is in line with a study showing that pseudoviruses carrying the S protein of the beta variant are most resistant to neutralization by mAb and covalent plasma antibodies (30). Similar to what we found in the analysis of sera of persons after the second vaccination, the IgA response of infected persons is more variable than the IgG response. Four persons (C1, C4, C5 and C6) belonging to the IgG high responder group also had the most robust IgA antibody response. The IgA class antibodies in the sera of infected persons are always bound more strongly to Ramos cells expressing the WT than to those with a variant S protein, with the beta variant being less well recognized than the alpha variant.

**Table 2:** Demographic data of vaccinated patients.

Vaccinated Cohort	PV (days)	SC V1 (days)	SC V2 (days)	Sex	Age
V1	0	14	10	M	28
V2	4	10	10	M	31
V3	2	12	10	F	57
V4	0	10	10	F	52
V5	4	10	10	F	34
V6	5	12	15	F	73
V7	-1	12	10	F	47
V8	4	10	10	M	36
V9	-1	11	11	F	43
V10	5	12	15	M	79
V11	2	10	10	M	56
V12	0	11	13	M	39

Median Age: 45 +/- 16,19 years; M, male; F, female; PV, Prevaccination; V1, First Vaccination; V2, Second Vaccination; SC, Sample Collection.

V8 had SARS-CoV-2 infection before vaccination.

### Stability and Immune Recognition of Mutated RBD

**Figure 6:**
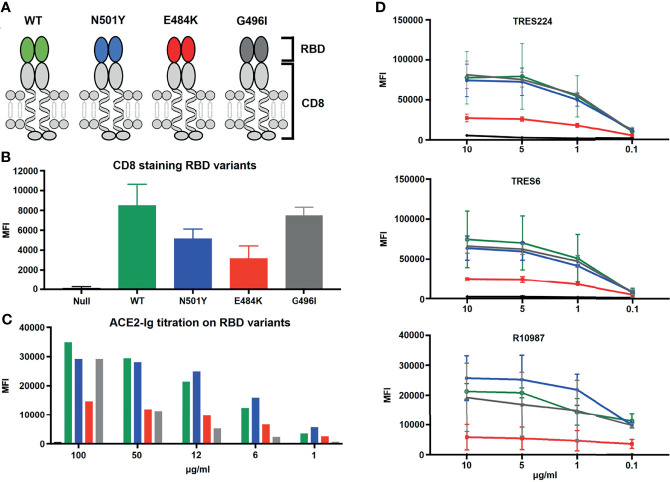
Influence of single amino acid exchange mutation in the RBD of the S protein on the expression and binding activity of the chimeric RBD-CD8 protein. **(A)** Schematic drawing of the RBD-CD8 protein with either an WT (green), or an N501Y (blue), E484K (red) or G496I (grey) mutated RBD sequence. **(B)** Flow cytometric analysis of the expression of the RBD-CD8 protein on Ramos cells stained with anti-mouse CD8 antibodies. **(C)** Titration of the ACE2-Ig binding to the four indicated Ramos cell populations. **(D)** Titration of mAb TRES224, TRES6 and R10987 binding to Ramos cells carrying the indicated RBD-CD8 proteins. As negative control we used non-transfected Ramos cells (black). The mean values of three different experiments are shown.

We next wanted to learn more about how a single amino acid exchange mutation in the RBD of the S protein influences the expression of the RBD-CD8 chimeric protein as well as its recognition by soluble ACE2-IgG or anti-RBD antibodies. For this, we introduced in the RBD-CD8 construct the N501Y and the E484K point mutations characteristic for the alpha and beta variant, respectively (**Figure 6A**). In addition, we introduced a glycine to isoleucine (G496I) amino acid exchange predicted to increase the interaction surface between the RBD and the ACE2 receptor (31). Ramos cells expressing the different RBD-CD8 constructs were barcoded and first tested for the chimeric RBD-CD8 protein expression by using anti-CD8 antibodies (**Figure 6B** and **Supplementary Figure 3**). This experiment showed that the N501Y and E484K mutant RBD-CD8 constructs are less expressed on Ramos cells than the WT or G496I mutated construct. Apparently, the two former mutations introduced some instability into the RBD of SARS-CoV-2 that is recognized by the quality control mechanism in the endoplasmic reticulum of Ramos B cell. We next exposed the four RBD-CD8-expressing Ramos-null cells to different concentrations of ACE2-Ig and analyzed them for receptor binding (**Figure 6C**).

**Figure 7:**
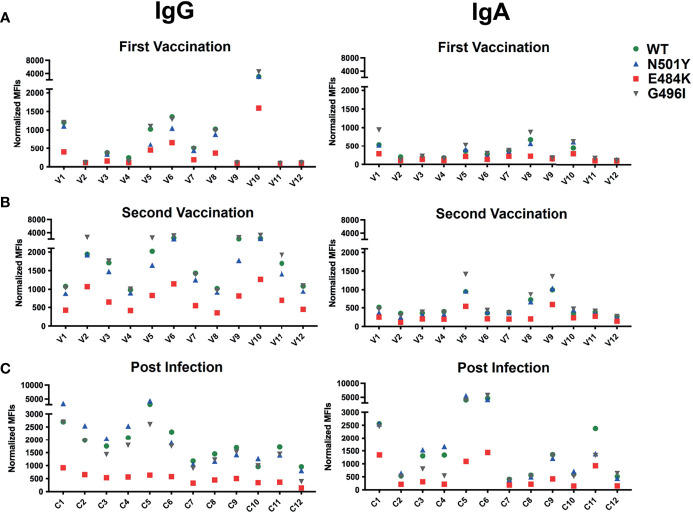
RBD binding activity of the sera of 12 persons vaccinated with the BioNTech/Pfizer mRNA vaccine after the primary **(A)** and secondary **(B)** vaccination doses and of 12 patients with severe COVID-19 disease **(C)**. Shown is the IgG (left panel) and IgA (right panel) antibody response of the sera diluted 1:100 and analyzed for binding to Ramos cells expressing RBD-CD8 protein with either a WT (green), or an N501Y (blue), E484K (red) or G496I (grey) mutated RBD sequence. Shown are normalized MFI values.

Interestingly, we found that, despite its lower expression, the N501Y mutant RBD-CD8 is bound better by ACE2-Ig than the WT RBD-CD8 construct, whereas Ramos cells expressing the G496I and E484K mutant RBD-CD8 constructs are less well bound by ACE2-Ig. This finding is in line with a biolayer interferometry study of ACE binding to RBD mutants (32). The three RBD-specific mAb (TRES224, TRES6 and R10987) bind to a similar extent to Ramos cells expressing the WT, N501Y, or G496I mutant RBD-CD8 proteins, whereas those cells expressing the E484K mutant RBD-CD8 constructs are poorly bound by these antibodies (**Figure 6D**). This study suggests that E484K is an immune escape mutation of the S protein. This notion is supported by BSFA of the immune sera of the 12 BioNTech/Pfizer vaccinated persons of our study groups (**Figure 7A, B**). RBD-specific IgG produced during the secondary response of the 12 vaccinated persons binds to a similar extent to Ramos cells carrying the WT, N501Y or G496I RBD-CD8 but to a lesser extent to those expressing the E484K mutant RBD-CD8 constructs. The analysis of the immune sera of the 12 infected persons with COVID-19 reveals a similar picture (**Figure 7C**). The RBD-specific IgA response showed a clear difference between the sera from the vaccinated and those of the SARS-CoV-2-infected group. Only 3 of the 12 BioNTech/Pfizer-vaccinated persons developed some RBD-specific IgA antibodies after the boost. In contrast, most infected persons showed an RBD-specific IgA response that was relatively high in three (C1, C5 and C6) of the infected COVID-19 patients (right panel **Figure 7**). However, as most IgA antibodies are present in mucosal tissues it is not clear how accurately blood IgA antibody levels represent the total IgA response.

## Discussion

We show here that retrovirally transfected Ramos-null B cells can stably express WT or mutated variants of the S proteins and chimeric proteins carrying only the RBD of the SARS-CoV-2 viruses. In combination with a color-coded barcoding method, this feature allowed us to compare different S-proteins or RBD mutants in their binding to either soluble ACE2-Ig molecule, specific mAb or antibodies of different classes in the sera of vaccinated or infected persons. Ramos cells are human B cells and are expected to express the trimeric S proteins with a glycosylation pattern and a membrane orientation found on viral particles emerging from human infected cells. The transfected Ramos cells share these features with HEK293T and Jurkat cells currently used in a coronavirus spike flow cytometric assay (16–18). An advantage of our system is that we use a retroviral transfection system to produce the native form of different S proteins on the surface of Ramos cells. In addition, retroviral vectors are efficiently and randomly integrated into many different gene loci of a transfected cell and thus generate a heterogenous population of transgene-expressing cells.

Furthermore, by using an IRES-GFP vector, we can to some extent correlate transgene with GFP expression. In this way, we can monitor the interaction of a specific binding reagent to Ramos cells carrying low, medium or high amounts of native S proteins on their surface. In addition, by using the color-coded barcoding method BSFA, we can combine up to 4 different Ramos cells in one test tube and expose them to the same binding reagent at a given concentration. In this way, we can directly compare the interaction of WT and mutant S proteins with either the soluble ACE2 receptor or specific antibodies. The BSFA can easily be adapted to test humoral immune responses against new SARS-CoV-2 variants and has the potential of high throughput of antibody screening and evaluation in a time-saving fashion.

Like other RNA viruses, SARS-CoV-2 can rapidly generate mutations during its expansion in an infected person (14). Thus, during the corona pandemic, several SARS-CoV-2 variants have emerged that became dominant in different world regions (33). Successful SARS-CoV-2 variants can be classified as either fitness and/or immune escape mutants (6). The former mutants infect and propagate themselves more efficiently in target cells, whereas the human immune system poorly recognizes the latter mutants. Our BSFA study found that Ramos cells expressing the full-length S proteins of the alpha and beta variant of SARS-CoV-2 are better bound by ACE2-Ig and by anti-RBD mAbs. This finding suggests that the variant S proteins are either more stably expressed on the Ramos cell than the WT S protein or resume a conformation with a more accessable RBD. In the case of the alpha variant, we provide direct evidence for a stronger ACE2 binding as the N501Y mutated RBD-CD8 chimera is less well expressed yet better recognized by the ACE2-Ig reagent. This is in line with data from a biolayer interferometry study (32). An unexpected finding of our study was that a single point mutation in the RBD reduces the expression of the RBD-CD8 chimera on transfected Ramos cells. Thus, these mutations seem to have an impact on the stability of the whole domain. In the full-length S protein, the RBD is either in a closed or a more open conformation, with only the open conformation able to bind to the ACE2 target (28). Hereto, the RBD amino acid point mutations selected by the alpha and beta variant may change not only the stability of the RBD but also the close/open equilibrium of the S protein and thus enable the variant virus to attach more readily to and infect its target cell.

With our BSFA approach, we can evaluate the quality of a coronavirus antibody response in terms of its specificity towards WT and variant S proteins and its target, namely epitopes within or outside the RBD structure. As most anti-RBD antibodies block the binding of the virus to the ACE2 entry receptor, they are likely to have neutralizing activity (12). Furthermore, with BSFA, we can analyze different classes of antibodies for these criteria. The evaluation of the humoral anti-SARS-CoV-2 immunity of the BioNTech/Pfizer-vaccinated group clearly showed an improved IgG response directed against the S protein and the RBD after the boost. Thus, two rounds of vaccinations are required for the efficiency of this vaccine. The IgG response of boosted persons was similar to that of patients with severe COVID-19 disease. However, the 50% high responder of the latter group developed more antibodies against the alpha variant. The humoral immunity towards the beta variant of SARS-CoV-2 was always lower in line with the finding that beta is an immune escape variant (30). A striking difference was seen in the IgA response between these groups. The S protein-specific IgA response did not improve after the second vaccination, and only 3 of the 12 vaccinated persons produced some anti-RBD antibodies after the boost. In contrast, 7 of the 12 COVID-19 patients had high IgA class anti-RBD antibodies titers in their serum.

In summary, our data show that our assay is a valuable tool to evaluate the humoral response of different immunoglobulin classes to either the vaccine or a SARS CoV-2 infection with either the wild-type or the mutant form of this virus.

## Data Availability Statement

The raw data supporting the conclusions of this article will be made available by the authors, without undue reservation.

## Ethics Statement

The study was approved by the Ethical Committee of the University of Freiburg (EK-Freiburg no 210/20 and 315/10). The patients/participants provided their written informed consent to participate in this study.

## Author Contributions

NV, cloned the expression vector and conducted the flow cytometric assay. YO, conducted the flow cytometric assay, developed the barcoding protocol and analysed the data. FB-B, helped with the cloning. JY, designed the expression vectors and the barcoding protocol. KR, provided the ACE-Ig construct. MT, provided the pVAX1-SARS2-S plasmid. SS, SF, HE and RV collected the human sera and organized the biobank. H-MJ, generated and provided the anti-S monoclonal antibodies. MR, designed and supervised the study. RV and MR wrote the manuscript. All authors contributed to the article and approved the submitted version.

## Funding

This project was funded in part by the German Research Foundation (Deutsche Forschungsgemeinschaft [DFG]) through TRR130 (project2 to MR), part of this work was supported by DFG 394523286, the Helmholtz association to KR, and the GRK1660, “NaFoUniMedCovid19” (FKZ: 01KX2021) - COVIM and the Bayerische Forschungsstiftung and the Kastnerstiftung to H-MJ, and the Bayerisches Staatsministerium für Wissenschaft und Kunst.

## Conflict of Interest

The authors declare that the research was conducted in the absence of any commercial or financial relationships that could be construed as a potential conflict of interest.

## Publisher’s Note

All claims expressed in this article are solely those of the authors and do not necessarily represent those of their affiliated organizations, or those of the publisher, the editors and the reviewers. Any product that may be evaluated in this article, or claim that may be made by its manufacturer, is not guaranteed or endorsed by the publisher.

## Supplementary Material

Supplementary Figure 1 Serum IgG (left) and IgA (right) response in 12 persons before receiving the fisrt dose of the BioNTech/Pfizer mRNA vaccine. Evaluation of the binding to Ramos cells expressing either the WT (green), the alpha variant (blue) or the beta variant (red) S protein. **(A)** or Ramos cells expressing RBD-CD8 protein with either a WT (green), or an N501Y (blue), E484K (red) or G496I (grey) mutated RBD sequences **(B)**. Shown are normalized mean fluorescence intensity (MFI) values.

Supplementary Figure 2 ELISA study for IgG-class anti-S1 antibodies in the sera of 12 persons vaccinated with the BioNTech/Pfizer mRNA vaccine and tested before (black), after the first (orange) or the secondary vaccination (green). Shown are the measure values related to the calibrator (measured value/calibrator).

Supplementary Figure 3 Flow cytometric analysis of the expression of the RBD-CD8 variants on Ramos Null cells stained with anti-mouse CD8 antibodies, mAb TRES224, serum IgG of the individuals C1 and V7.
