# Supplementary figures and images for: A Barcoded Flow Cytometric Assay to Explore the Antibody Responses Against SARS-CoV-2 Spike and Its Variants
Source: Front Immunol. 2021 Sep 23;12:730766. doi: 10.3389/fimmu.2021.730766 (PMC8496935; doi:10.3389/fimmu.2021.730766)

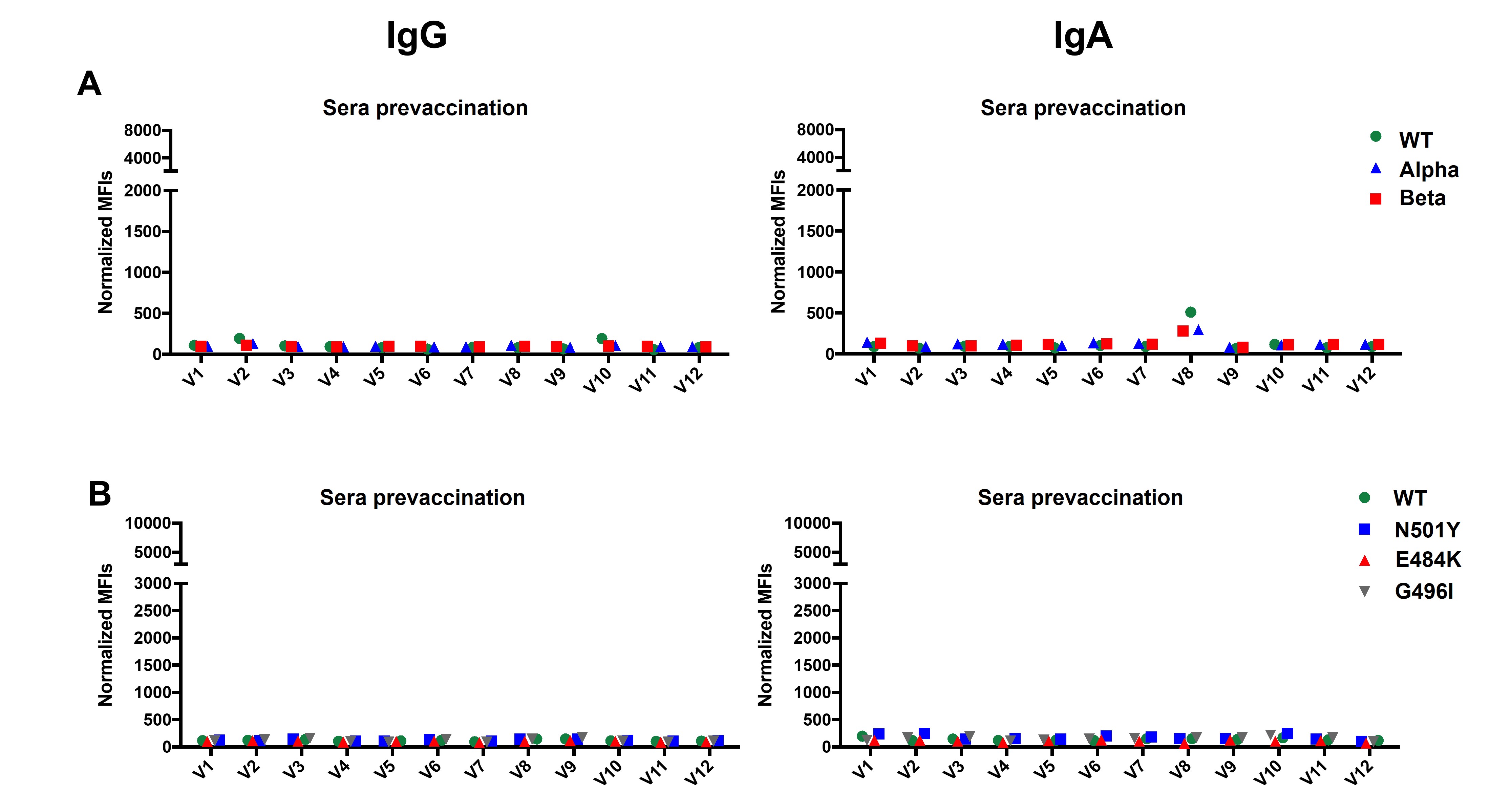

Supplement: Supplementary Figure 1 — Serum IgG (left) and IgA (right) response in 12 persons before receiving the fisrt dose of the BioNTech/Pfizer mRNA vaccine. Evaluation of the binding to Ramos cells expressing either the WT (green), the alpha variant (blue) or the beta variant (red) S protein. (A) or Ramos cells expressing RBD-CD8 protein with either a WT (green), or an N501Y (blue), E484K (red) or G496I (grey) mutated RBD sequences (B). Shown are normalized mean fluorescence intensity (MFI) values. [file Image_1.jpeg]

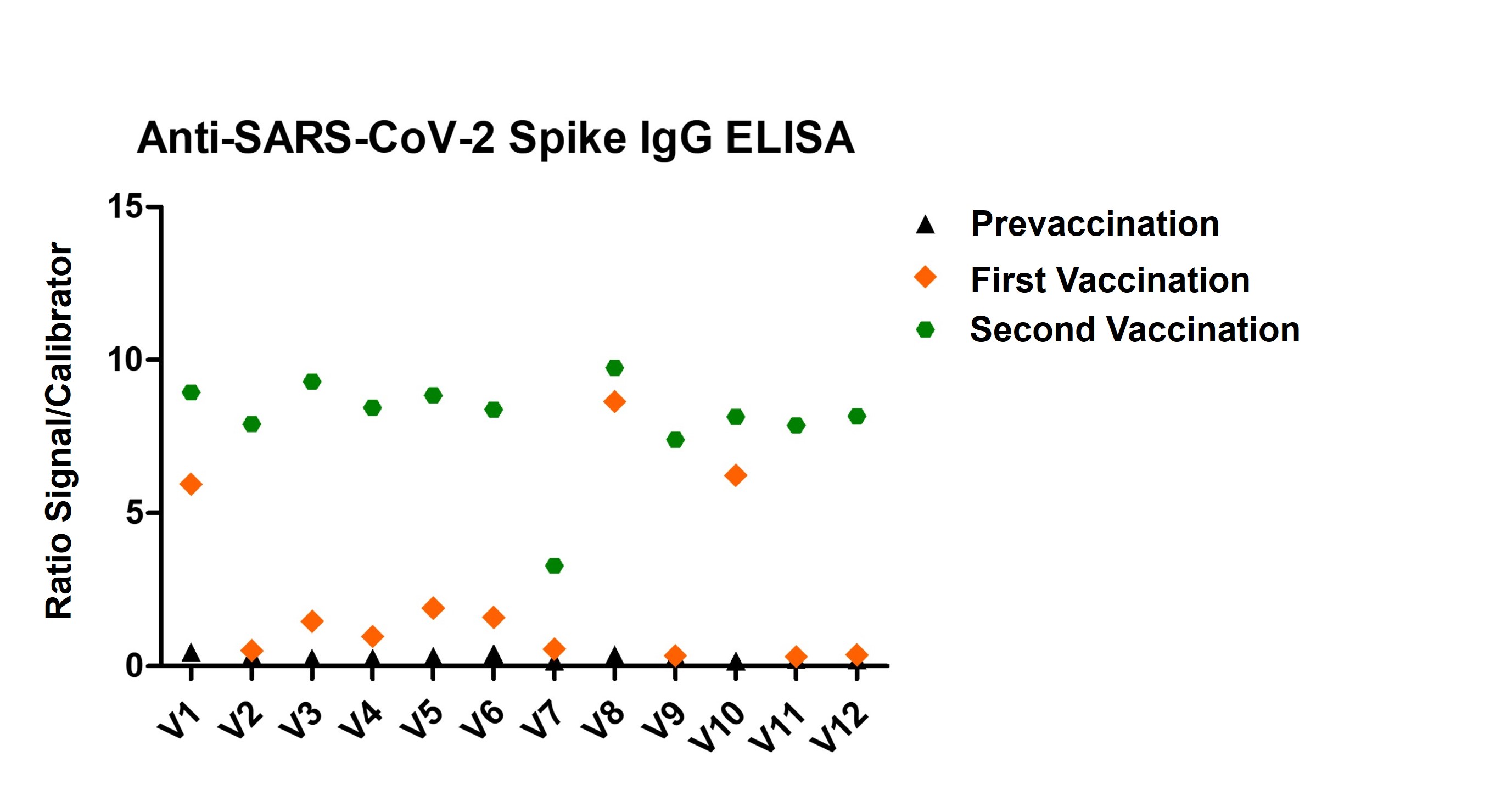

Supplement: Supplementary Figure 2 — ELISA study for IgG-class anti-S1 antibodies in the sera of 12 persons vaccinated with the BioNTech/Pfizer mRNA vaccine and tested before (black), after the first (orange) or the secondary vaccination (green). Shown are the measure values related to the calibrator (measured value/calibrator). [file Image_2.jpeg]

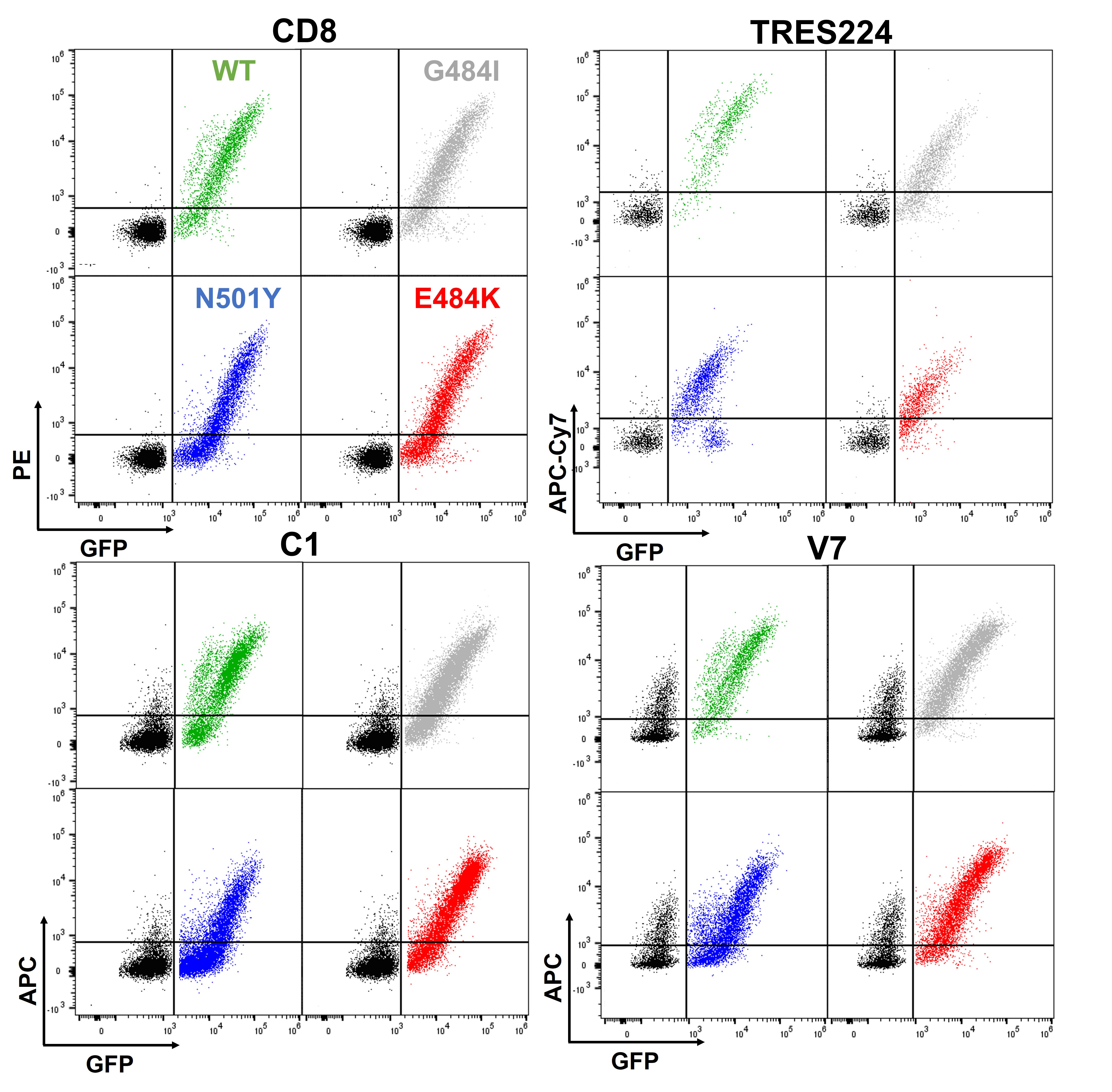

Supplement: Supplementary Figure 3 — Flow cytometric analysis of the expression of the RBD-CD8 variants on Ramos Null cells stained with anti-mouse CD8 antibodies, mAb TRES224, serum IgG of the individuals C1 and V7. [file Image_3.jpeg]
